# Whole genome analysis and cold adaptation strategies of *Pseudomonas sivasensis* W-6 isolated from the Napahai plateau wetland

**DOI:** 10.1038/s41598-023-41323-x

**Published:** 2023-08-30

**Authors:** Lingling Xiong, Yanmei Li, Hang Yu, Yunlin Wei, Haiyan Li, Xiuling Ji

**Affiliations:** 1 Faculty of Life Science and Technology, Kunming University of Science and Technology, Kunming, China; 2 Medical School, Kunming University of Science and Technology, Kunming, China; 3 Yunnan International Joint Laboratory of Research and Development of Crop Safety Production on Heavy Metal Pollution Areas, Kunming, China

**Keywords:** Ecology, Microbiology

## Abstract

Microbial communities of wetlands play key roles in the earth’s ecology and stability. To elucidate the cold adaptation mechanisms of bacteria in plateau wetlands, we conducted comparative genomic analyses of *Pseudomonas sivasensis* and closely related lineages. The genome of *P. sivasensis* W-6, a cold-adapted bacterium isolated from the Napahai plateau wetland, was sequenced and analyzed. The genome length was 6,109,123 bp with a G+C content of 59.5%. Gene prediction yielded 5360 protein-coding sequences, 70 tRNAs, 24 gene islands, and 2 CRISPR sequences. The isolate contained evidence of horizontal gene transfer events during its evolution. Two prophages were predicted and indicated that W-6 was a lysogen. The cold adaptation of the W-6 strain showed psychrophilic rather than psychrotrophic characteristics. Cold-adapted bacterium W-6 can utilize glycogen and trehalose as resources, associated with carbohydrate-active enzymes, and survive in a low-temperature environment. In addition, the cold-adapted mechanisms of the W-6 included membrane fluidity by changing the unsaturated fatty acid profile, the two-component regulatory systems, anti-sense transcription, the role played by *rpsU* genes in the translation process, etc. The genome-wide analysis of W-6 provided a deeper understanding of cold-adapted strategies of bacteria in environments. We elucidated the adaptive mechanism of the psychrophilic W-6 strain for survival in a cold environment, which provided a basis for further study on host-phage coevolution.

## Introduction

With the continuous improvement of sequencing technology, microbial genomic analysis has progressively enriched and expanded the content of the database. Based on the representative species in the system development between the comparative analysis of genes and gene families, comparative genomics for constructing the genetic map in system development reveals the origin and function of the genes and gene families, and its mechanism of complication and diversification in the process of evolution.

Cold environments are widely distributed and represent about 75% of the earth^1^. Cold-adapted microorganisms can be divided into two categories based on the growth temperature, psychrophilic and psychrotrophic microorganisms. They utilize a series of metabolic strategies to grow in low-temperature environments. It accumulates glycogen and gluconeogenesis as energy to help improve antifreeze capability^2–5^, maintaining the fluidity of cell membranes^6^, coping with oxidative stress caused by low temperature^7^, and the expression of molecular chaperones extracellular polysaccharides also played important roles^8^. In recent years, due to the application potential of low-temperature microorganisms and their products, studies on microbial genomic sequencing from cold environments have been reported^8,9^.

*Pseudomonas* spp*.* belong to the Gram-negative bacterium, mostly aerobic or facultative anaerobic, and widely distributed in nature. Until now, only 5 genomes of *P. sivasensis* have been sequenced based on NCBI GenBank, among which the BsEB-1 strain was the most completely sequenced. Among the genome size of all the *P. sivasensis* genomes published, the size was approximately 6.2 Mbps. The GC content of different *P. sivasensis* genomes ranged about 60%.

Glycogen is an important nutritional currency and energy source. Compared with other sugars, glucose is an optimal carbon source. Under different abiotic stresses, such as low temperature, nutrient deprivation, osmotic regulation, and pH maintenance, glycogen synthesis is one of the well-developed energy storage systems for bacteria to adapt and survive^10^. Glycogen metabolism is a complex interaction network that involves multiple genes and pathways^11^. The most important five enzymes are involved in glycogen metabolism: ADP-glucose pyrophosphorylase (GlgC), glycogen synthase (GlgA), glycogen branching enzyme (GlgB), glycogen phosphorylase (GlgP), and glycogen debranching enzyme (GlgX)^12^. Bacteria develope a passive energy-saving strategy, such as nutrient deprivation with slow glycogen degradation. Metabolism of maltodextrin is related to glycogen metabolism, and 4-glucanotransferase (MalQ) medicates the recycling of maltose to maltodextrins^13^.

Trehalose is a disaccharide of two D-glucose molecules linked by a glycosidic linkage and plays an important role in protecting bacteria against a range of stresses^14^. It is well known that trehalose protects microbes, such as *Saccharomyces cerevisiae* and *Escherichia coli* from desiccation^15,16^. Abiotic stresses impose selection pressure on bacterial environmental durability and may activate some pathways or express specific genes, which might help bacterial survival. Five trehalose-related pathways are involved in bacteria: TPS/TPP (OtsBA), TreYZ, TreS, TreP, and TreT biosynthetic pathways^14^. Trehalose and glycogen metabolism are related through the treS-pep2-glgE-glgB pathway^17^. Generally, there are multiple synthesis pathways in a single bacterium which indicates the importance of compatible modes.

In this study, whole genome sequencing of *P. sivasensis* W-6, a cold-adapted bacterium isolated from the Napahai plateau wetland, was performed to provide evidence for how the strain W-6 responded in a cold environment. It provides information on new microorganisms and their adaptability in a low-temperature environment.

## Results and discussion

### Growth conditions and identification

The growth of the strain W-6 was measured at different temperatures of 4, 10, 15, 20, 25, 30, and 37 °C. It can be seen that strain W-6 could grow at the range from 4 to 30 °C with the optimal growth temperature 15 °C. Then, the growth curve of strain W-6 was made by detecting OD_600_ at 15 °C (Fig. S1). The strain W-6 entered the logarithmic growth period after 10 h and reached the stable period at 40 h. So, strain W-6 was a psychrophilic rather than psychrotrophic bacterium. Based on 16S rRNA gene amplification (1501 bp, NCBI accession number MF949058), DNA sequencing, and BLASTn in GenBank, the isolate was identified as *P. sivasensis*.

### Characteristic analysis of genome

**Figure 1:**
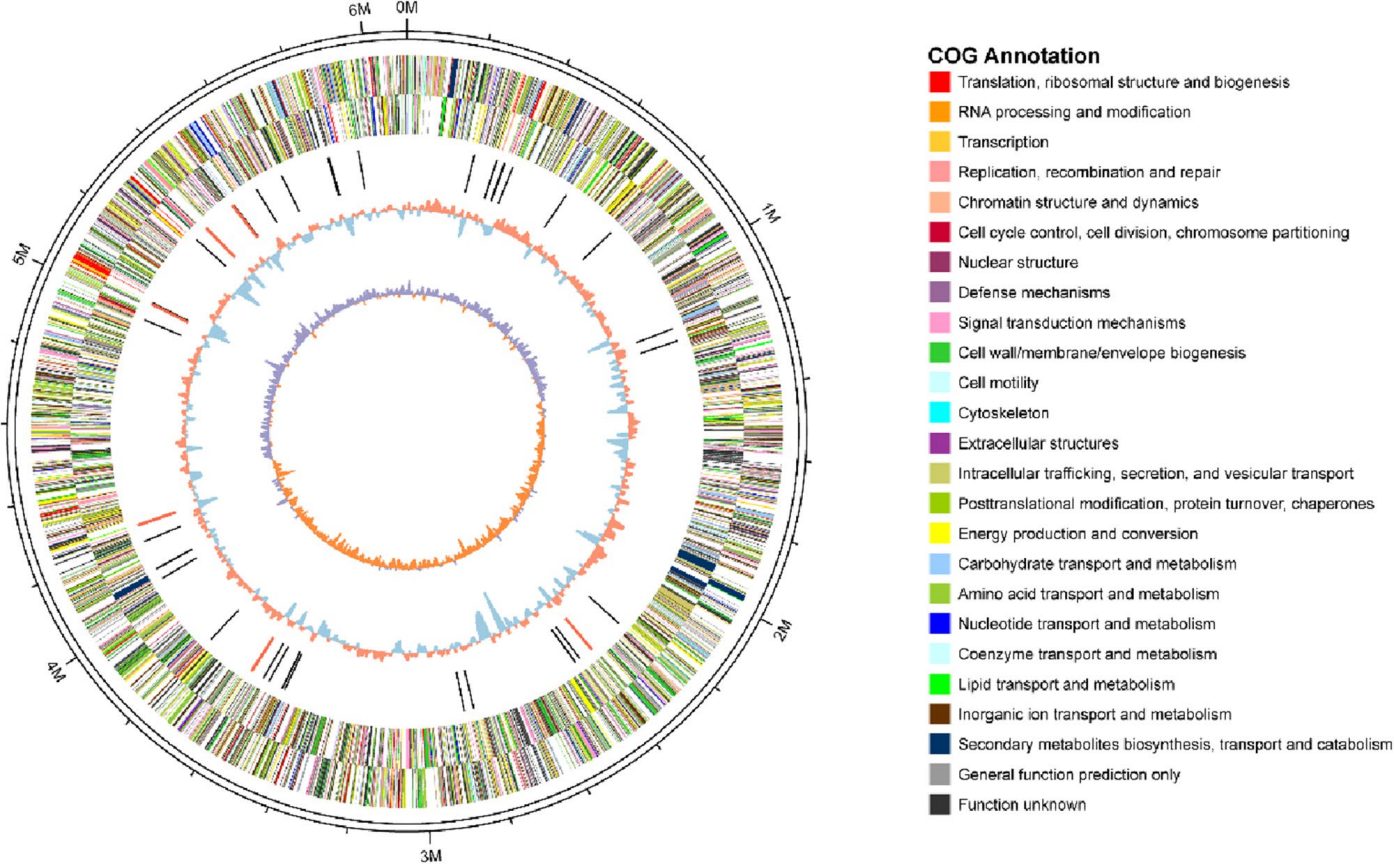
Genomic circle map of *Pseudomonas sivasensis* W-6. The outermost circle is the position coordinates of the genome sequence. From the outer circle to the inner circle are: positive strand gene, negative strand gene, Color indicates COG classification, ncRNA (black: tRNA, red: rRNA), GC content (red > mean value, blue < mean value), GC skew (purple < 0, orange > 0).

After obtaining the original data by sequencing, it was filtered and corrected, and then the sequence was spliced and assembled. One scaffold was acquired, assembled, and had a total length of 6,109,123 bp with a G+C content of 59.5% (Table S1). The genomic circle map of *P. sivasensis* W-6 was shown in Fig. 1. Table S2 showed that the total genome length and G+C content were more comparable to those of other strains of *P. sivasensis*. In addition, 19 rRNAs and 70 tRNAs were obtained from *P. sivasensis* W-6.

### Function annotation of genes in genome

**Figure 2:**
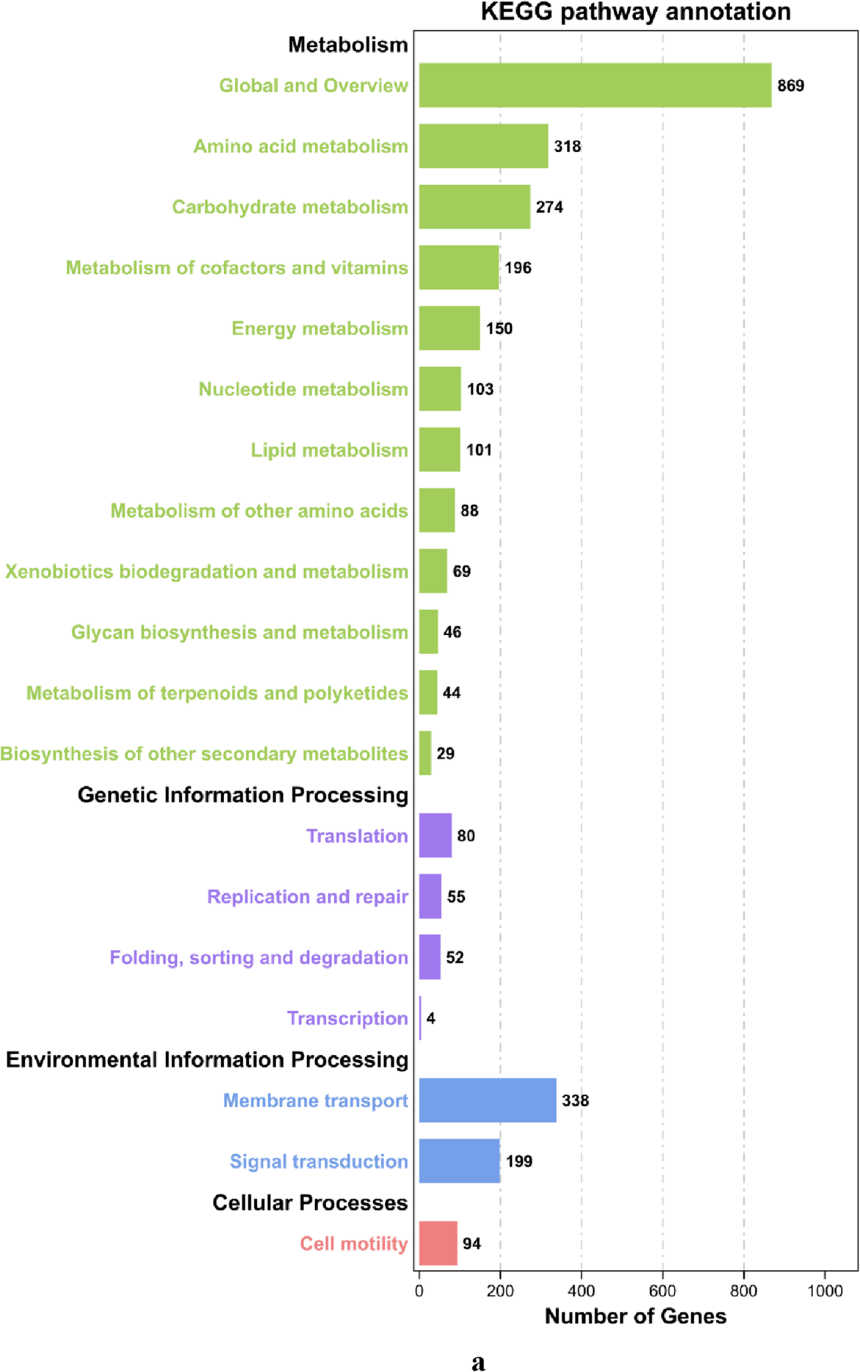

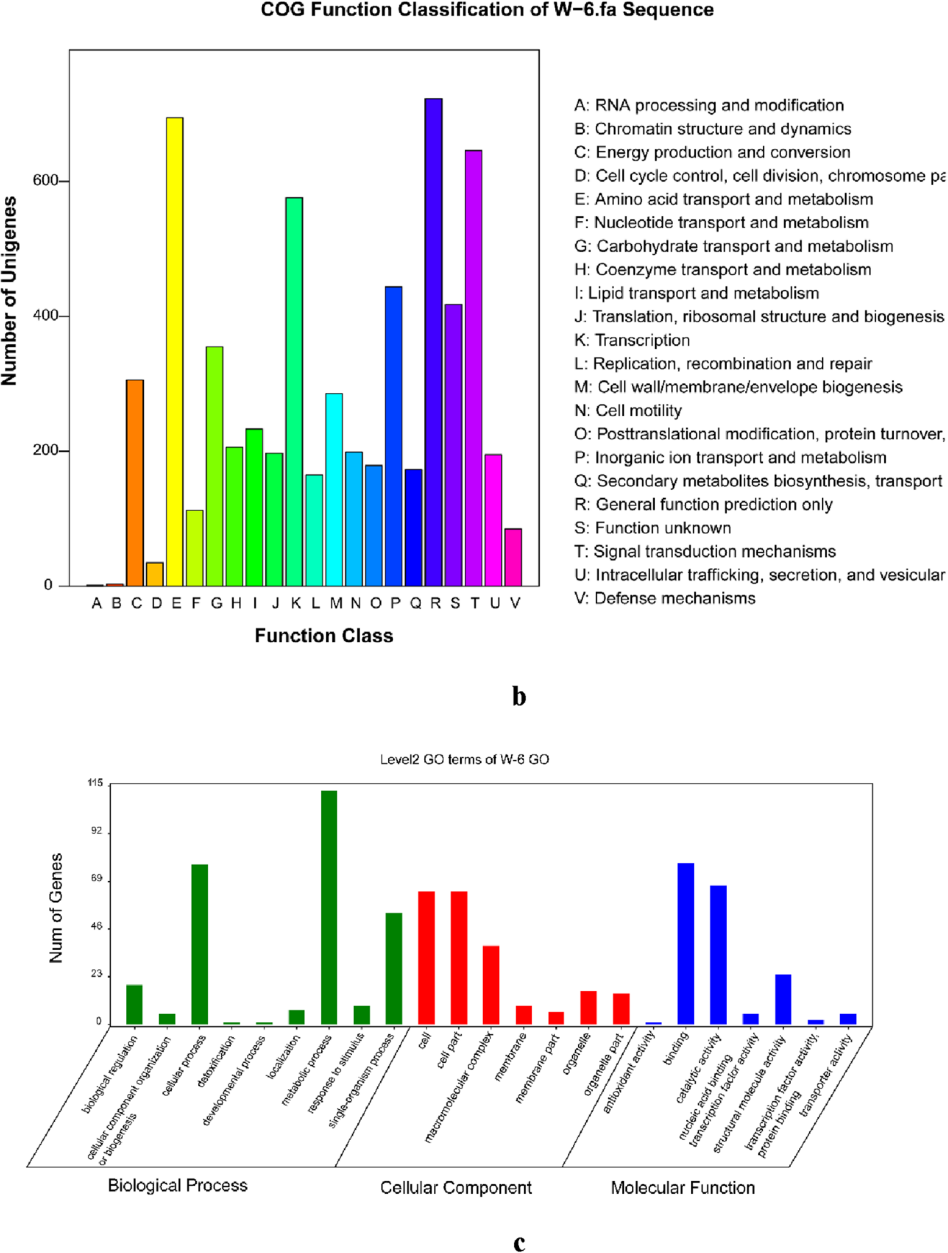
Function categorization of genes of the *Pseudomonas sivasensis* W-6. (**a**) Based on the KEGG database. The different colors represent different pathways and the numbers in the diagram represent the number of genes in such pathways. The green color represents KEGG pathways related to cell metabolism, purple color represents KEGG pathways related to genetic information processing, blue color represents KEGG pathways related to environmental information processing, and pink color represents KEGG pathways related to represents cellular processes. (**b**) COG function classification of *Pseudomonas sivasensis* W-6 genome. The different colors represent different COG function classification and the numbers in the diagram represent the number of genes in such pathways. (**c**) GO function annotation chart of W-6. The horizontal axis represents the functional classification, which is displayed in different colors, and the vertical axis represents the number of predictive genes contained in each functional classification.

According to the KEGG annotation information, 3063 genes were annotated in W-6 with 115 pathways, which can be classified into five categories: metabolism, genetic information processing, environmental information processing, cellular processes, and tissue systems (Fig. 2a). The number of metabolic pathway genes was 1477, accounting for 48.23% of the annotated genes. Many secondary metabolic biosynthesis, microbial metabolism in different environments, antibiotic biosynthesis, ABC transporter system, and two-component system were also annotated with 349, 289, 261, 241, and 199 genes, respectively. ABC transporter proteins are widely present in microorganisms. They play an important role in the nutrient uptake of ions, monosaccharides, amino acids, phospholipids, peptides, polysaccharides, and proteins. The two-component system regulates amino acid metabolism and mediates the stress response to the external environment. In addition, carbon metabolism and bacterial secretion system-related pathways had a large of genes.

Through the COG functional annotation of the W-6 genome (Fig. 2b), it showed that the W-6 strain had the most proteins in the R class (general function prediction, 723), followed by the E class (amino acid transport and metabolism, 695), the T class (signal transduction mechanism, 646), the L class (replication, recombination, and repair, 576), and the P class (inorganic ion transport and metabolism class, 444). These classes accounted for 49.48% of the total W-6 proteins, which was a significant proportion of all proteins.

The GO database has three major categories: molecular function, cellular component, and biological process of genes, respectively (Fig. 2c). The number of genes in the cellular component was 856 and 64 for both cellular and cytosolic functions. The number of genes in the molecular function category was the lowest, with only 546, but 78 performed the binding function. In sum, the genes involved in metabolic processes, cellular and cellular components, and binding functions were the most numerous, indicating that these functions played a key role in W-6.

In the functional annotation of W-6 encoded proteins, we found that most of the proteins in the W-6 strain were amino acid transport and metabolism, signal transduction mechanism, replication, recombination and repair, and inorganic ion transport and metabolism, which accounted for 49.48% of the total W-6 proteins, which was a significant proportion of all proteins. Not only as the basic units of proteins and amino acids but also function in the free form in cells. These free amino acids were either precursors or intermediate of metabolism or storage forms of free ammonia to eliminate their toxic effects on the body, whereas the role of these proteins in bacteria was not clear and needed to be further explored.

Similar annotation results were obtained in the GO functional annotation of the W-6 genome. Up to 113 functional genes belonging to biological process genes were involved in metabolic processes. Metabolic pathways accounted for 48.23% of the total genes annotated in the pathway annotation. Thus, the largest proportion of protein functions in W-6 were metabolism-related proteins.

### Comparative genomic analysis of *P. sivasensis* W-6

**Figure 3:**
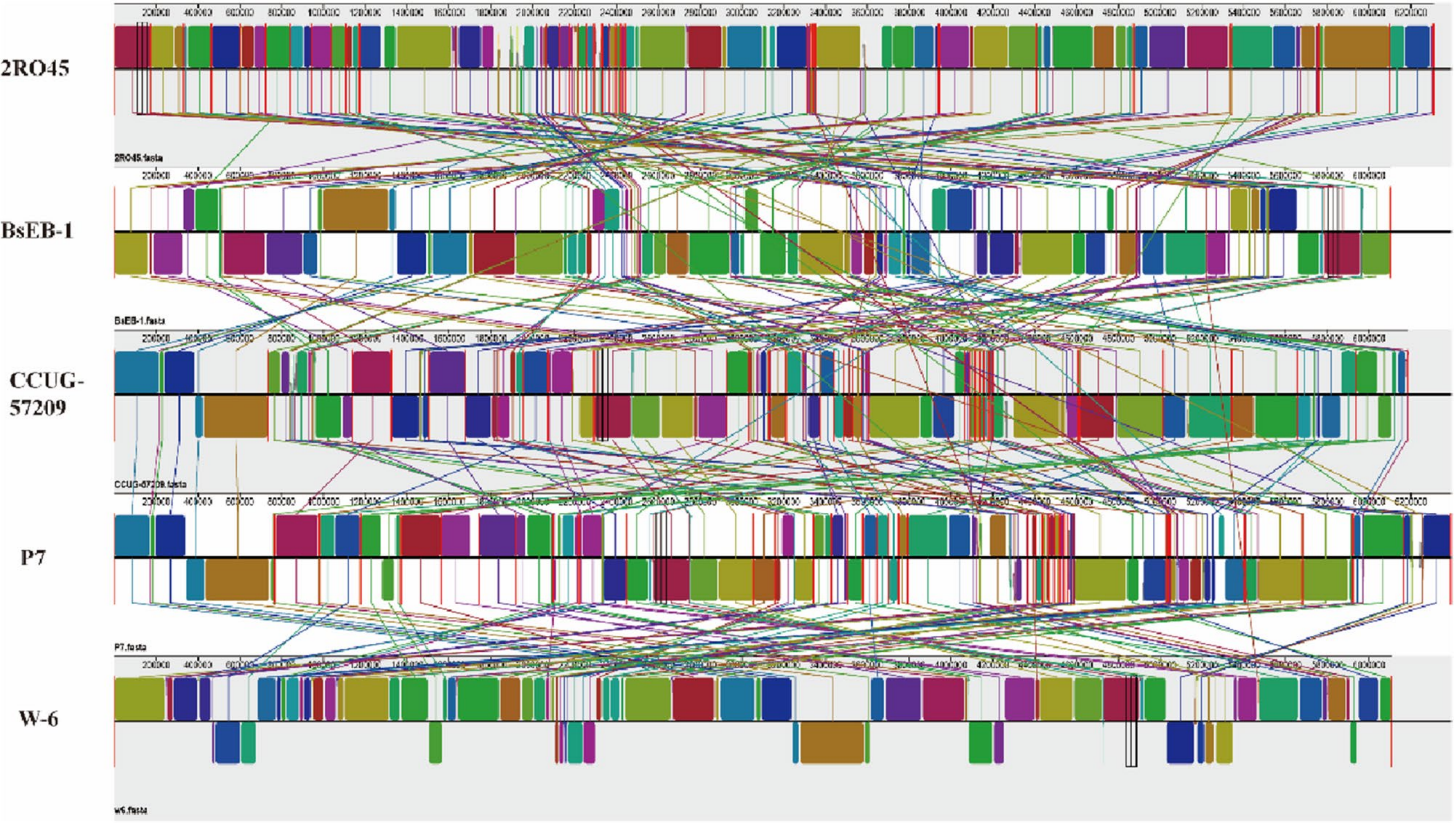
Collinearity analysis of five *Pseudomonas sivasensis*. Mauve visualization of locally collinear blocks (LCBs) identified the five *Pseudomonas sivasensis* genomes. Each contiguously colored region is a locally collinear block. LCBs below a genome’s center line are in the reverse complement orientation relative to the reference genome. The colored blocks include similar regions between the bacterial genomes. Red vertical bars demarcate intrachromosomal boundaries. The Mauve enables users to zoom in on regions of examine the local rearrangement structure.

The genomic information on five *P. sivasensis* strains was shown in Table S2. The collinearity analysis was performed by combining W-6 with the nucleic acid sequences of the other four *P. sivasensis* strains in two combinations (Fig. 3).

**Figure 4:**
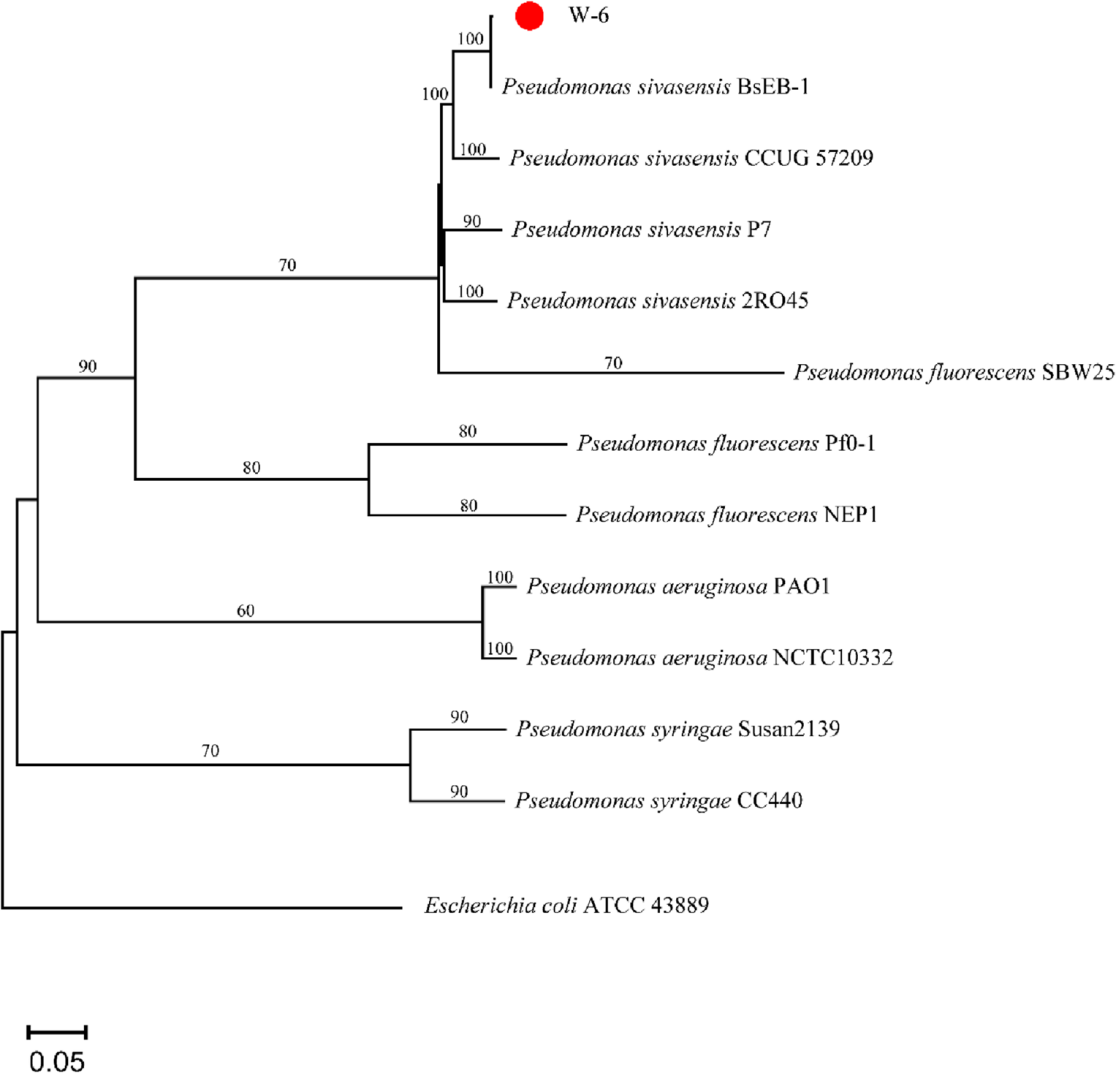
Phylogenetic tree of W-6 strain. Phylogenetic tree showing the relationships of the *P. sivasensis* W-6 and others strains. Red dot means W-6 strain.

W-6 showed stronger collinearity with four *P. sivasensis* strains (2R045, BsEB-1, CCUG-57209, and P7), both forward and reverse collinearity. The differences between W-6, 2R045, BsEB-1, CCUG-57209, and P7 genomes were small, although these bacteria come from different habitats. At the same time, CheckM^18^ was used to evaluate the quality and completeness of W-6 and obtained a completeness of 99.66%, contamination of 0.08, and Strong Heterogeneity of 0. FastANI (default parameter) was used to calculate the bacterial complete genome average nucleotide identity (ANI) of W-6 with the reference P7 strain (99.04%). The values of digital DNA-DNA hybridization (dDDH) of W-6 with the reference strains BsEB-1 and P7 were 100% and 93%, respectively. Average nucleotide identity (ANI, 88.3679%) and dDDH (62.7%) were detected between strain W-6 and *P. fluorescens* SBW25. We could see that the comparative genome result was much higher than the common ANI values of 92–97%. It showed the ANI of W-6 with the closely related species was 99.04%. ANI and dDDH closely reflect the traditional concept of relatedness, but ANI values do not represent genome evolution, due to the variation of the compared genomes. However, this study classified the strain and allocated it through a whole genome phylogenetic tree. A phylogenetic tree was constructed between W6 strains and the other genomes (Fig. 4). We could see that the W-6 was closest to the BsEB-1. It was consistent with the result of dDDH. Thus, the W-6 strain belonged to *P. sivasensis*.

### Mobile genetic elements

Mobile genetic elements play a crucial role in genome evolution, conferring bacterial adaptation to various environmental conditions. Mobile genetic elements also contribute to horizontal gene transfer (HGT).

Prophages are integrated viral forms in bacterial genomes. Prophage sequences propagate vertically to progeny together with bacterial cell division, contributing to the interstrain genetic variability and adaptive evolution of bacteria. Two prophage loci were predicted in the chromosome, phiW-6–1 (41,297 bp, positions 1,571,724–1,613,020) and phiW-6-2 (38,126 bp, positions 1,603,523–1,641,648). Eleven and seventeen phage-related genes were identified in these regions, respectively (Table S3). The DNA synthesis genes were found in two prophage loci, indicating that these were not replication-defective (Table S4). The phiW-6-1 was similar to VW6S (94%) and contained 45 ORF (gene_1403-gene_1447), including Rz protein (*gp 24*), cell wall endolysin (*gp 25*), tail filament assembly protein (*gp 27*), tail filament domain protein (*gp 28*), and tail filament protein (*gp 30*). Prophage phiW-6-2 was similar to phage ФAH14a (87%) and contains 53 ORF, including ФAH14a head morphology protein (AH14a_p63), portal protein (AH14a _ p62), terminal enzyme (AH14a _ p61), capsid protein (AH14a_ p66), transcription regulator (AH14a_p06), and DNA methyltransferase (AH14a_p05). There were 11 ORFs (gene_1437-gene_1447) overlapping between phiW-6-1 and phiW-6-2, such as head morphogenesis proteins, terminal enzymes, single-stranded DNA binding proteins, and structural proteins. Horizontally acquired DNA, also known as mobile genetic elements, includes transposons, plasmids, and prophage, among which prophage is the most important factor^19^. During the bacterial life cycle, some genes encoded by prophage are active and closely related to host resistance, pathogenicity, antibiotic resistance, virulence or metabolism, biofilm formation, and stress^20–23^. Prophage could protect the host from double infection and confer new resistance on the host^24^. Prophages are diverse, evading immunity and supporting the survival and dominance of lysogenic phages, which may be important drivers in shaping microbial ecosystems.

The transposon prediction of the W-6 genome identified only one transposon, located at 1,861,323–1,861,742. It indicated that the genetic plasticity of strain W-6 might not be determined by intragenomic rearrangements.

CRISPRs are a component of many bacterial genomes, and CRISPRs function in the interference pathway to preserve genome integrity. In the W-6 chromosome, two CRISPRs were detected. CRISPR1 had one spacer and CRISPR2 had three spacers.

### Drug resistance gene annotation of *P. sivasensis* W-6

Currently, due to antibiotic ubiquity and misuse, the problem of bacterial resistance has become a serious issue^25^. It will be of great use for the analysis of bacterial resistance isolated in the natural ecological environment. A total of 56 resistance genes were found in the W-6 strain by predictive annotation in both Antibiotic Resistance Genes Database (ARDB) and Comprehensive Antibiotic Resistance Database (CARD) databases (Table S5), which were classified into seven categories: Efflux pumps, Fluoroquinolones, Polypeptides, β-Lactams, Polyphosphate, Peptide antibiotics, and Elfamycin. Among them, there were 46 resistance genes in Efflux pumps, accounting for 82.14% of the total number of resistance genes (10 resistance genes). Efflux pump genes had a considerable proportion in W-6 and may show a crucial role. It may be related to the environment W-6 was located, and the exact relationship needs to be explored.

### CAZymes-encoding genes in *P. sivasensis* W-6

**Figure 5:**
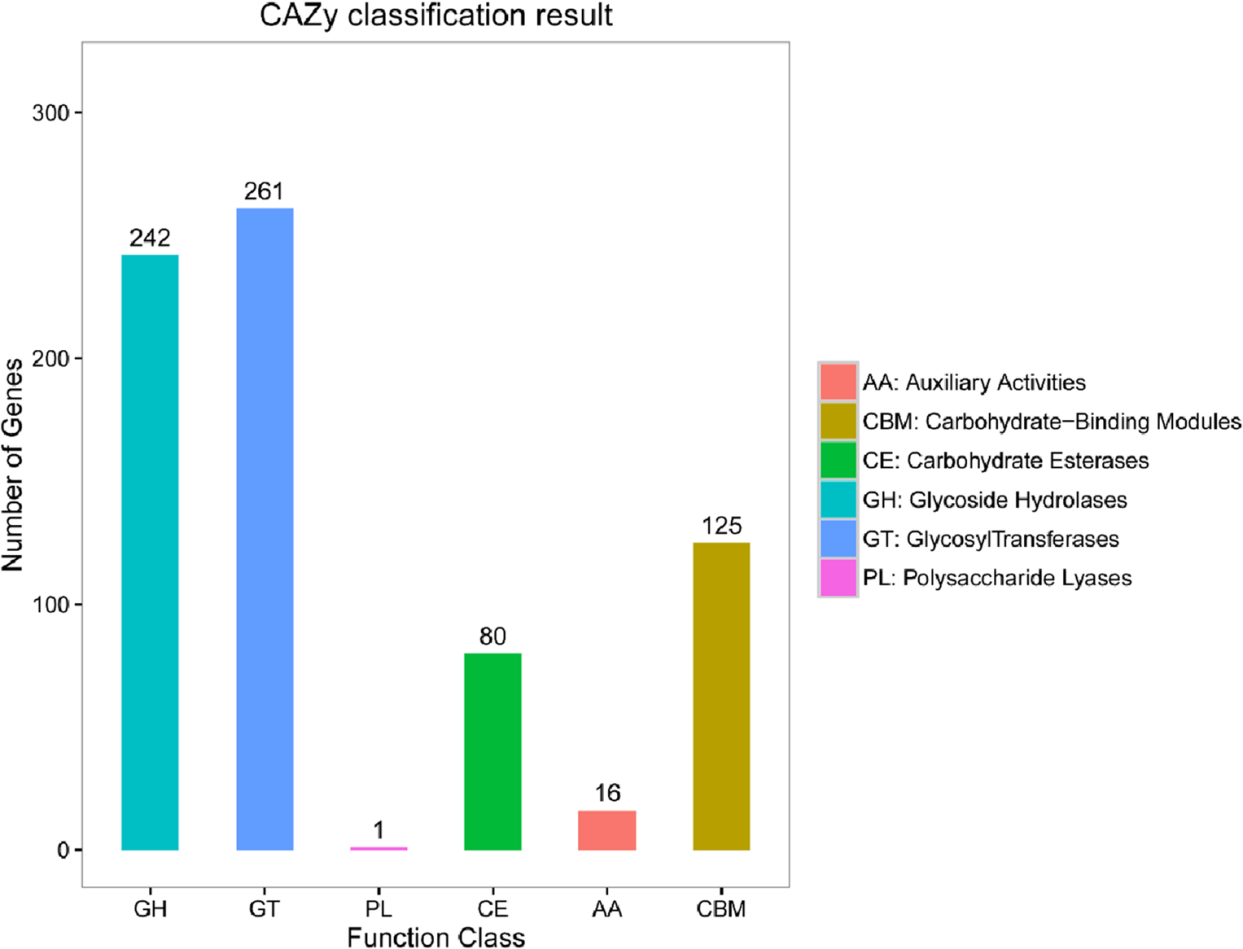
Gene distribution of CAZymes in W-6 strain. The horizontal axis represents the classification of enzymes (different colors represent different kinds of enzymes), and the vertical axis represents the number of genes contained in this classification.

Among the identified protein-encoding genes in *P. sivasensis* W-6, 725 were significantly annotated and classified into Carbohydrate active enzymes** (**CAZymes**)** groups (GH, GT, CE, AA, CBM, and PL). It provided insight into the carbohydrate utilization mechanisms of W-6. Figure 5 depicted the 242 Glycoside Hydrolases (GHs), 261 Glycosyl Transferases (GTs), 80 Carbohydrate Esterases (CEs), 16 Auxiliary Activities (AAs), 125 Carbohydrate-Binding Modules (CBMs), and one Polysaccharide Lyases (PL). These genes were related to amino acid transport, transcription, carbohydrate transport, and energy production/conversion, which suggested that the strain W-6 utilized CAZymes for energy storage and carbohydrate metabolism. Most bacteria rely on catabolizing carbohydrates and then obtain energy. Such as GH13 families that could break down starch or cellulose, and trehalose-related genes (OtsA) are involved in energy storage.

*Shigella* sp. PAMC28760 could adapt and survive in cold environments through glycogen metabolism^26^. *Bacillus* sp. TK-2 possessed cold evolution adaptability through CAZymes genes related to the degradation of polysaccharides containing cellulose and hemicellulose^9^. The disruption of the glycogen metabolism pathway compromised *E. coli* survival in a cold environment^10^. *Arthrobacter* sp. PAMC26654 utilized polysaccharide or carbohydrate degradation as a source of energy to adapt and survive in cold environments, especially CAZymes, which are active at low temperatures^27^. Genomic analyses have revealed genomic information and evolutionary insights about different strains and species from cold environments. However, compared with eukaryotes, the characteristics of glycogen metabolism in prokaryotes are still not well-studied, and the metabolism of low-temperature microorganisms is not well understood^28^. The analysis of the W-6 complete genome suggested that glycogen and trehalose metabolisms were associated with CAZymes genes. The CAZymes may have important significance in low-temperature adaptation, especially for glycogen and trehalose metabolism-related genes^9,10,23,24^.

### Glycogen metabolism and the trehalose pathway in *P. sivasensis* W-6

We analyzed the metabolism pathways of glycogen metabolism and trehalose metabolism in 14 *Pseudomonas* strains. Based on the composition of GH, GT, and other main enzymes in 14 selected genomes, similarities of different environments were analyzed. The genes of W-6 contained were different from those of other *Pseudomonas* strains (*GlgP, GlgC, TreA,* and *TreR*). Compared with the other 14 strains, it was unique to W-6. Therefore, W-6 had slightly different pathways for energy acquisition or polysaccharide degradation than other’s *Pseudomonas* spp. (Table S6). Among the genes related to glycogen metabolism and trehalose metabolism, *glgC*, *otsA*, *otsB*, and *treT* genes were missing in all 14 strains. Thus, most *Pseudomonas* spp. missed OtsA/B, whereas encoded TreS and TreY/Z^29^. Among these 14 genomes, there were partial genes overlap between W-6 and the other 13 strains, such as *GlgX, GlgA, GlgB, OtsA, TreS, TreZ, TreY, TreP,* and *SugB*, but there were also differences. which could indicate that glycogen and trehalose metabolic pathways may exist in W-6 differently from the 13 strains. It may be one of the key factors for W-6 to adapt to a low-temperature environment.

**Figure 6:**
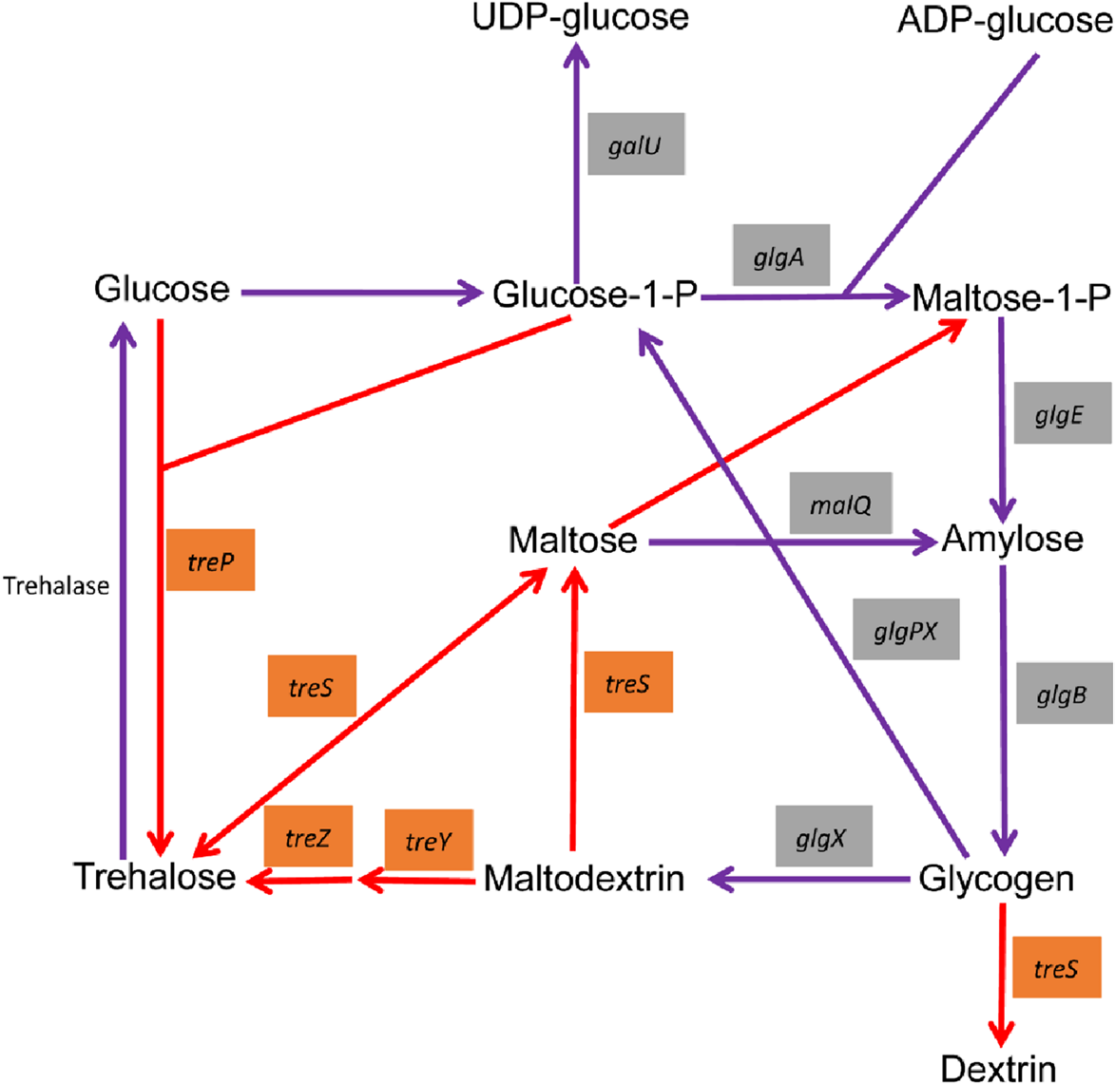
Glycogen and trehalose metabolic pathway in W-6. The gray box represents the glycogen metabolism related genes, and the orange box represents the related genes of trehalose metabolism. The purple line represents the glycogen metabolic pathway, and the red line represents trehalose metabolism pathway.

The relationship between glycogen and trehalose metabolic pathways in bacteria is shown in Fig. 6. There are three main pathways of glycogen metabolism. The most common glycogen metabolism pathway in bacteria involves the *glgC* gene^10^, while the *galU* gene is generally more common in fungi^11^. The metabolic pathways of trehalose are well known in bacteria, for example, a defense strategy involving trehalose accumulation. Trehalose and glycogen were highly accumulated in *Propionibacterium freudenreichii* under cold conditions^30^. OtsBA, TreYZ, and TreS existed in *Arthrobacter* sp. PAMC25564^29^, *Bacillus* sp. TK2^9^, *P. freudenreichii*^30^ and *Mycobacterium* sp.^31^, but OtsA/B missing in most *Pseudomonas* spp.^29^. Five trehalose metabolic pathways have been reported in some bacteria^14^, such as TPS/TPP, TreY-TreZ, TreP, TreS, and TreT pathways, facilitating survival in cold environments. Trehalose was essential for *E. coli* viability at low temperatures^32^. Based on the CAZy gene annotation, *P. sivasensis* W-6 contained most of the genes involved in these two metabolic pathways, except for the *glgC*, *otsA*, *otsB*, and *treT* genes. Gene *glgC* encodes a key enzyme in the glycogen metabolic pathway, so the glycogen metabolic pathway is commonly involved in bacteria with *glgC*, but it is not present in W-6. The *galU* genes generally involved in glycogen metabolism in fungi, but the *galU* genes present in the W-6 genome, thus inferring that glycogen metabolism in W-6 may be different from those in general bacteria. There were four main trehalose metabolism pathways in *S. acidocaldarius,* including TPS/TPP, TreY-TreZ, TreH, and TreT pathways^33^. However, W-6 did not contain *otsA, otsB*, and *treT* genes, so three trehalose metabolism pathways were involved in TreY-TreZ, TreS, and TreP pathways in W-6. This may be a unique feature of W-6 in the adaptation to low-temperature environments. At present, the roles of trehalose and glycogen are currently poorly understood in *Pseudomonas* spp.

Thus, the metabolic pathways of glucose, trehalose, and maltose were interconnected. It provides an understanding of survival adaptation in the cold environment by comparative analysis of glycogen metabolism and trehalose pathway from different *Pseudomonas* spp.

### Others cold adaptation strategies

Membrane fluidity by changing the unsaturated fatty acid profile is a universal strategy to adapt to a cold environment^34^. Eight genes (*bdcA*, *bacC*, *fadB*, *fadJ*, *tesA*, *tesB*, *fabC*, and *fabG*) were identified as involved in the synthesis of unsaturated fatty acids, and these genes were most likely important for maintaining the membrane fluidity of W-6 under cold stress. Glutathione maintains cell redox homeostasis and protects membrane lipids from the oxidative stress induced by cold stress^35^. Glutathione synthase (GshB, W-6-4258), two key genes encoding glutathione peroxidase (Gpx2, W-6-2237), and glutathione reductase (Gor, W-6-1098), involved in the cycle of glutathione, were identified in the W-6 genome, indicating that glutathione may facilitate psychrotolerant of the strain W-6. The cold shock protein (CSP) CspA, glutathione peroxidase, and superoxide dismutase were found in W-6 and *Pseudomonas* sp. HS6^36^. However, the cold adaptation mechanisms of *Pseudomonas* sp. HS6 attributed to CSP and amino acid usage^36^. Typical CSPs also played in *P. fluorescens* PF08^37^ and KUIN-1^38^. Polyhydroxyalkanoate (PHA), involved in carbohydrate metabolism, was an important factor in the growth of *Pseudomonas* sp. 14-3 strain at low temperatures^39^. By comparative analysis of metabolism pathways from different *Pseudomonas* spp., we learned more about cold adaptation strategies.

Two-component regulatory systems are responsible for bacterial survival in cold environments^40^. A two-component regulatory system is composed of a sensor kinase and a response regulator. In the W-6 genome, 199 pairs of sensor kinase and response regulator were found, which may act as a multifunctional sensory to control numerous cold-responsive genes as well as responses to osmotic, salt, and oxidative stress^41^.

The *sfsA* (W-6-4762) was the cold-induced regulatory gene in the W-6 strain, belonging to the DNA-binding transcriptional regulator and regulating the sugar catabolism. The production of RNA helicases (*deaD, hrpB, hrpA, pcrA, rapA, rhlE, rhlB, dbpA, uvrD,* and *ywqA*) also could be induced by low temperatures, then prevent the formation of structured nucleic acids.

Anti-sense transcription may lead to RNA secondary structure inaccuracy, low efficiency, slow speed, and false fidelity of transcription and translation under cold stress^41^. In bacteria, *nusG* is a co-factor of the Rho transcriptional terminator and could diminish genome-wide anti-sense transcription combination with histone-like nucleoid-structuring protein (H-NS) and Rho-dependent transcriptional terminators^42^. Therefore, the expression of *nusA/nusB/nusG* (W-6-4739, W-6-4562, and W-64489, respectively) may benefit W-6 to silence the anti-sense transcription for survival in a cold environment.

The *rpsU* gene (W-6-4435) in the W-6 genome encoding the 30S ribosomal subunit protein, may play an important role in cold adaptation. As can be reported in *Synechocystis*, the *rpsU* gene was induced tenfold under cold stress^43^. The ribosome chaperone trigger factor (Tig, W-6-0528) in W-6 may help with early folding and prevent misfolding and aggregation of proteins. The SmpB protein (W-6-4717) in W-6 is needed to rescue ribosomes stalled on defective messages^43^.

In sum, the analysis of the W-6 genome suggested that cold-adapted bacterium W-6 had glycogen and trehalose metabolism pathways associated with CAZyme genes, and they were used as energy sources to adapt and survive in cold environments. The metabolic pathways of glucose, trehalose, and maltose were interconnected. It provides an understanding of survival adaptation in a cold environment of W-6. Adaptations of the W-6 strain to low temperatures also were conferred by membrane fluidity by changing the unsaturated fatty acid profile, the two-component regulatory systems, anti-sense transcription, the role played by *rpsU* genes in the translation process, etc.

## Materials and methods

### Growth conditions, DNA extraction and identification

To elucidate the cold adaptation mechanisms of bacteria in plateau wetlands, some cold-adapted bacteria were obtained. *P. sivasensis* W-6 was isolated from the water sample collected from the Napahai plateau wetland (27° 53′ 36′′ N 99° 38′ 24′′ E) in Yunnan province, China, on 23 May 2019. The strain W-6 was incubated at different temperatures at 4 °C, 10 °C, 15 °C, 20 °C, 25 °C, 30 °C, and 37 °C on sterile LB solid media. Bacterial universal primer set 27F-AGAGTTTGATCCTGGCTCAG, 1492R-GGTTACCTTGTTACGACTT was used for 16S rRNA amplification and molecular identification^44^. The amplified fragments were subjected to DNA sequencing and using BLASTn to determine the bacterial species. PCR amplification conditions with 30 amplification cycles were as follows: 95 °C for 4 min, 95 °C for 40 s, 50 °C for 45 s, and 72 °C for 90 s. For genomic DNA isolation, the *P. sivasensis* W-6 strain was cultured in a sterile LB liquid medium at 15 °C with 150 rpm. High-quality genomic DNA of the W-6 was extracted using Biospin bacteria genomic DNA extraction Kit and verified by gel electrophoresis on 0.7% agarose. The W-6 strain has been deposited to the China General Microbiological Culture Collection Center with number CGMCC 1.62087.

### Genome sequencing, assembly and annotation

The genome sequencing libraries were prepared and then sequenced on PacBio RSII. The Library preparation protocol was as follows, the extracted DNA sample was segmented, followed by DNA damage repair and terminal repair. Next, the splice was connected, followed by Exo III and Exo IV digestion, and then the DNA was further purified to remove the shorter library and adapter dimers. At the same time, to improve the sequencing quality, it is necessary to perform Blue Pippin (BP) fragment selection on the library to remove short fragment library molecules. FastQC^45^ performs quality control and filter conditions to remove reads with a length of less than 100 and those with an average quality value of less than 0.80. The quality-filtered reads (Q ≥ 100) were assembled with HGAP^46^ software based on PacBio data, and spliced and assembled through overlap. Scaffolding utilized the alignments between contigs and reads to determine the relative orientation and order of contigs, ultimately producing longer scaffolds, and facilitating further analysis and interpretation of genomic data^44^. The ORFs were predicted with Glimmer^47^ software and identified using BLAST with NCBI nr and Pfam^48^ (https://pfam.xfam.org) as reference databases. The functional gene annotation was performed by NCBI nr, Swiss-Prot^49^, COG^50^, and KEGG^51^ databases. The tRNA and rRNA were predicted with tRNA scan-SE 1.21^52^ and RNAmmer^53^, respectively. Clustered, regularly-interspaced short palindromic repeats (CRISPRs)/Cas genes were identified by CRISPR/Cas finder online tool (http://crispr.i2bc.paris-saclay.fr/Server/). Transposon PSI (http://transposonpsi.sourceforge.net/) was employed to predict transposable factors. The prophage was predicted using Prophage Hunter (https://pro-hunter.genomics.cn/). CAZymes was annotated using automated carbohydrate-active enzyme annotation web server-dbCAN Meta server (http://bcb.unl. edu/dbCAN2/blast.php). The virulence genes were predicted using the Virulence Factor of Bacterial Pathogen database (VFDB) and Virulence Finder v2.0 (http://cge.cbs.dtu.dk/services/VirulenceFinder/). The pattern of antibiotic resistance was investigated with Comprehensive Antibiotic Resistance Database (CARD). According to the Antibiotic Resistance Genes Database (ARDB) (ardbAnno1.0)^54^, we used RGI to research antibiotic resistance-related gene information. The genome sequence was deposited in GenBank.

### Comparative genome and genome synteny analyses

The whole genome sequence of the W-6 strain was deposited in NCBI GenBank with the accession number CP058533.1. CheckM^18^ was used to evaluate the quality and completeness of the W-6. ANI and dDDH values were calculated using fastANI^55^ and GGDC (https://ggdc.dsmz.de/faq.php). The genome phylogeny of *P. sivasensis* W-6 was analyzed with MEGA X^56^ software for the phylogenetic tree construction with the Maximum Likelihood method. For comparative genome analysis, the sequences of the phylogenetically closest type strain with the available genome of *P. sivasensis* W-6 and well-characterized genomes of *P. sivasensis* were chosen (Table S1), which were retrieved from the NCBI database. The comparative genome features with *P. sivasensis* W-6 were listed in Table S2. Mauvey^57^ software was used for genome analysis and visualization.

### Supplementary Information

Supplementary Information.

## Data Availability

The datasets generated and analysed during the current study are available in the NCBI GenBank database, [https://www.ncbi.nlm.nih.gov/nuccore/CP058533].
