# Supplementary material for: Whole genome analysis and cold adaptation strategies of Pseudomonas sivasensis W-6 isolated from the Napahai plateau wetland
Source: Sci Rep. 2023 Aug 30;13:14190. doi: 10.1038/s41598-023-41323-x (PMC10468529; doi:10.1038/s41598-023-41323-x)
Supplement: Supplementary file 1 — Supplementary Information. [file 41598_2023_41323_MOESM1_ESM.docx]

**Table S1 Genomic feature of *Pseudomonas sivasensis* W-6**

| Feature | W-6 |
| --- | --- |
| scaffold  Genome Size (Mb)  GC content (%)  Genes (total)  CDSs (total)  Genes (coding)  CDSs (with protein)  Genes (RNA)  tRNA  Prophage (bp): phiW-6-1  Prophage (bp): phiW-6-2 | 1  6.1  59.5  5583  5490  5365  5365  93  70  41,297  38,126 |

**Table S2 Basic information on strains of *Pseudomonas sivasensis***

| Name | GenBank accession | Genome size (bp) | No. of CDS | Habitat |
| --- | --- | --- | --- | --- |
| W-6 | CP058533 | 6,109,123 | 5,583 | Napahai plateau wetland, Yunnan, China, |
| BsEB-1 | CP090029.1 | 6,101,651 | 5,485 | Root, China: Kunming, Yunnan |
| P7 | JAAOWU000000000.1 | 6,393,295 | 5,757 | Turkey: Central Anatolia, Kayseri |
| CCUG 57209 | NZ_JAAOWV000000000.1 | 6,183,640 | 5,633 | Sweden |
| 2RO45 | NZ_JAOAQN000000000.1 | 6309106 | 5,730 | canola rhizosphere  Poland: Ostroda |

**Table S3** **Prediction of prophages**

| Candidate ID | Start | End | Length | Subject ID | Subject Name | Identity | Query coverage | E value | Bitscore |
| --- | --- | --- | --- | --- | --- | --- | --- | --- | --- |
| Candidate_1 | 1,571,724 | 1,613,020 | 41,297 | MF975720.1 | Pseudomonas phage VW-6S | 94% | 13% | 0 | 7591 |
|  |  |  |  | KU708004.1 | Pseudomonas phage phiAH14a | 89% | 38% | 0 | 3238 |
|  |  |  |  | KT968831.1 | Pseudomonas phage YMC11/02/R656 | 73% | 16% | 0 | 1283 |
|  |  |  |  | KY271399.1 | Klebsiella phage 5 LV-2017 | 71% | 3% | 1.33E-171 | 610 |
|  |  |  |  | KJ507100.1 | Pseudomonas phage phiPSA1 | 73% | 4% | 6.46E-144 | 518 |
| Candidate_2 | 1,603,523 | 1,641,648 | 38,126 | KU708004.1 | Pseudomonas phage phiAH14a | 87% | 26% | 0 | 3110 |
|  |  |  |  | KY271399.1 | Klebsiella phage 5 LV-2017 | 71% | 3% | 1.23E-171 | 610 |
|  |  |  |  | KJ507100.1 | Pseudomonas phage phiPSA1 | 73% | 3% | 5.96E-144 | 518 |
|  |  |  |  | KT968831.1 | Pseudomonas phage YMC11/02/R656 | 72% | 3% | 8.84E-142 | 511 |
|  |  |  |  | MF975720.1 | Pseudomonas phage VW-6S | 80% | 1% | 2.22E-111 | 410 |

**Table S4** **Candidate_1 and Candidate_2 annotation of prophages**

| Candidate_1 | | | Candidate_2 | | |
| --- | --- | --- | --- | --- | --- |
| Gene ID | Protein length (aa) | NCBI_nr | Gene ID | Protein length (aa) | NCBI_nr |
| gene_1403 | 215 | putative PhoH family protein | gene_1437 | 167 | virion protein |
| gene_1404 | 75 | heat shock protein | gene_1438 | 194 | hypothetical protein BPABA456_00280 |
| gene_1405 | 526 | diguanylate cyclase | gene_1439 | 321 | major capsid protein |
| gene_1406 | 336 | zinc-containing alcohol dehydrogenase superfamily protein | gene_1440 | 174 | hypothetical protein |
| gene_1407 | 304 | tape measure protein | gene_1441 | 361 | putative head protein, SPP1 gp7 family |
| gene_1408 | 69 | putative DNA polymerase | gene_1442 | 472 | portal protein |
| gene_1409 | 424 | error-prone lesion bypass DNA polymerase V | gene_1443 | 433 | putative large terminase subunit |
| gene_1410 | 141 | protein umuD | gene_1444 | 153 | hypothetical protein BPPAER656_00810 |
| gene_1411 | 240 | hypothetical protein StPS1_gp24 | gene_1445 | 198 | hypothetical protein |
| gene_1412 | 574 | aerotaxis sensor receptor protein | gene_1446 | 146 | putative single-stranded DNA-binding protein |
| gene_1413 | 159 | hypothetical protein Sm_phiM9_122 | gene_1447 | 84 | structural protein |
| gene_1414 | 172 | lysozyme | gene_1448 | 51 | hypothetical protein C421010_055 |
| gene_1415 | 141 | putative endolysin | gene_1449 | 110 | holin |
| gene_1416 | 70 | hypothetical protein MAR_21 | gene_1450 | 320 | baseplate wedge initiator |
| gene_1417 | 127 | hypothetical protein | gene_1451 | 65 | hypothetical protein |
| gene_1418 | 383 | tail fiber protein | gene_1452 | 77 | hypothetical protein CPT_Moon231 |
| gene_1419 | 1188 | hypothetical protein BPPAER656_00480 | gene_1453 | 181 | hypothetical protein |
| gene_1420 | 220 | tail assembly protein | gene_1454 | 198 | protein NinG |
| gene_1421 | 116 | hypothetical protein mEpX2_019 | gene_1455 | 156 | hypothetical protein |
| gene_1422 | 186 | hypothetical protein AD45P1_00350 | gene_1456 | 261 | hypothetical protein |
| gene_1423 | 86 | major capsid protein | gene_1457 | 325 | hypothetical protein |
| gene_1424 | 89 | putative inhibitor of prohead protease | gene_1458 | 59 | hypothetical protein SLPG_00008 |
| gene_1425 | 254 | hypothetical protein BPPAER656_00610 | gene_1459 | 218 | prophage repressor |
| gene_1426 | 250 | hypothetical protein BPPAER656_00620 | gene_1460 | 77 | gp13 |
| gene_1427 | 112 | minor tail protein M | gene_1461 | 72 | #N/A |
| gene_1428 | 1116 | hypothetical protein BPPAER656_00640 | gene_1462 | 158 | hypothetical protein DA66_0110 |
| gene_1429 | 89 | hypothetical protein BPPAER656_00660 | gene_1463 | 107 | baseplate wedge subunit and tail pin |
| gene_1430 | 126 | hypothetical protein | gene_1464 | 83 | hypothetical protein PAEP54_00550 |
| gene_1431 | 218 | hypothetical protein BPPAER656_00680 | gene_1465 | 127 | LuxR family transcriptional regulator |
| gene_1432 | 216 | ORF001 | gene_1466 | 98 | hypothetical protein KWAN_272 |
| gene_1433 | 139 | hypothetical protein BPPAER656_00690 | gene_1467 | 98 | hypothetical protein EcP1_gp02 |
| gene_1434 | 218 | hypothetical protein BPPAER656_00710 | gene_1468 | 112 | major capsid protein |
| gene_1435 | 122 | hypothetical protein CPKG_00054 | gene_1469 | 105 | gp23 major head protein |
| gene_1436 | 65 | hypothetical protein | gene_1470 | 65 | tail length tape measure protein |
| gene_1437 | 167 | virion protein | gene_1471 | 64 | hypothetical protein PAEP54_00650 |
| gene_1438 | 194 | hypothetical protein BPABA456_00280 | gene_1472 | 268 | putative phage recombination protein |
| gene_1439 | 321 | major capsid protein | gene_1473 | 542 | hypothetical protein BA3_0032 |
| gene_1440 | 174 | hypothetical protein | gene_1474 | 80 | putative minor tail protein |
| gene_1441 | 361 | putative head protein, SPP1 gp7 family | gene_1475 | 156 | hypothetical protein |
| gene_1442 | 472 | portal protein | gene_1476 | 112 | gp169 |
| gene_1443 | 433 | putative large terminase subunit | gene_1477 | 153 | putative ATP-dependent DNA helicase |
| gene_1444 | 153 | hypothetical protein BPPAER656_00810 | gene_1478 | 694 | DNA cytosine methyltransferase |
| gene_1445 | 198 | hypothetical protein | gene_1479 | 38 | hypothetical protein SHANETTE_118 |
| gene_1446 | 146 | putative single-stranded DNA-binding protein | gene_1480 | 285 | hypothetical protein |
| gene_1447 | 84 | structural protein | gene_1481 | 32 | DNA cytosine methyltransferase |
|  |  |  | gene_1482 | 107 | portal protein |
|  |  |  | gene_1483 | 143 | hypothetical protein BMBtpLA_55 |
|  |  |  | gene_1484 | 71 | unnamed protein product |
|  |  |  | gene_1485 | 47 | CII decision making protein |
|  |  |  | gene_1486 | 62 | hypothetical protein SP19_10 |
|  |  |  | gene_1487 | 88 | lysozyme family baseplate assembly protein |
|  |  |  | gene_1488 | 85 | HNH endonuclease |
|  |  |  | gene_1489 | 397 | putative integrase |

**Table S5 Basic information of antibiotic resistance genes in W-6**

| Antibiotic resistant group | quantity | gene |
| --- | --- | --- |
| Efflux pumps | 46 | *abeM, acrB, acrD, adeA, adeJ, adeL, baeR, evgS, farB, macA, macB, mdsC, mdtB, mdtC, MexA, MexB, MexC, MexD, MexE, MexF, MexJ, MexK, mexM, mexN, MexV, MexW, msbA, MuxA, MuxB, MuxC, OpmB, OpmH, OprA, OprJ, OprM, OprN, PmpM, Pseudomonas aeruginosa CpxR, smeA, smeC, smeF, smeR, smeS, TriA, TriB, TriC* |
| Fluoroquinolones | 3 | *gyrB、gyrA、mfd* |
| Polypeptides | 2 | a*rnA、pmrF* |
| β- Lactams | 2 | PDC-5、PBP2 |
| Polyphosphate | 1 | *murA* |
| Peptide antibiotics | 1 | *mprF* |
| Elfamycin | 1 | *Streptomyces cinnamoneus* EF-Tu mutants |

**Table S6 Comparative analysis of predicted pathways for glycogen and trehalose metabolism in *Pseudomonas* strains**

|  |  |  |  | ***P. sivasensis*** | ***P. fluorescens*** | | | | | | ***P. aeruginosa*** | | | | | | | | |
| --- | --- | --- | --- | --- | --- | --- | --- | --- | --- | --- | --- | --- | --- | --- | --- | --- | --- | --- | --- |
| **Enzyme** | **CAZyme** | **EC number** | **Functions** | **W-6** | **SIK_W1** | **L111** | **SBW25** | **NEP1** | **WH6** | **R124** | **PPF-1** | **Pa127** | **LYSZa2** | **CCBH4581** | **VA-134** | | **PA1RG** | **BIM B-268** | **LYT4** |
| GlgP | GT35 | 2.4.1.1 | glycogen phosphorylase | + | - | - | - | - | - | - | - | - | - | - | | - | - | - | - |
| GlgX | CBM48+GH13_9 | 2.4.1.18 | 1,4-α-glucan debranching enzyme | + | + | + | + | + | + | + | + | + | + | + | | + | + | + | + |
| GlgA | GT5 | 2.4.1.21 | glycogen synthase | + | + | - | + | - | + | + | + | + | + | + | | + | + | + | + |
| GlgB | GT5 | 2.4.1.342 | glycogen branching enzyme | + | + | - | + | - | + | + | - | + | + | + | | + | + | + | + |
| GlgC |  | 2.7.7.27 | glycogen synthase, ADP-glucose transglucosylase | + | - | - | - | - | - | - | - | - | - | - | | - | - | - | - |
| GalU | GH0 | 2.7.7.9 | UDP-glucose pyrophosphorylase | - | - | - | - | - | - | - | - | - | - | - | | - | - | - | - |
| OtsA | GT20 | 2.4.1.15 | trehalose 6-phosphate synthase | + | + | - | + | + | + | + | + | + | + | + | | + | + | + | + |
| OtsB | GT20 | 3.1.3.12 | trehalose 6-phosphate phosphatase | - | - | - | - | - | - | - | - | - | - | - | | - | - | - | - |
| MalQ | GH77 | 2.4.1.25 | 4-α-glucanotransferase | - | - | - | - | - | - | - | - | - | - | - | | - | - | - | - |
| TreS | GH13_16 | 3.2.1.1 | maltose α-D-glucosyltransferase / α-amylase | + | + | - | + | + | + | + | + | + | + | + | | + | + | + | + |
| TreZ | GH13_10 | 3.2.1.141 | maltooligosyltrehalose trehalohydrolase | + | + | - | + | + | + | + | + | + | + | + | | + | + | + | + |
| TreY | GH13_26 | 5.4.99.15 | (1→4)-α-D-glucan 1-α-D-glucosylmutase | + | + | - | + | + | + | + | - | + | + | + | | + | + | + | + |
| TreA | CBM48 |  |  | + | - | - | - | - | - | - | - | - | - | - | | - | - | - | - |
| TreR |  |  |  | + | - | - | - | - | - | - | - | + | + | + | | + | + | - | + |
| TreP | GH32 |  | trehalose phosphorylase | + | + | + | + | - | + | - | - | - | - | - | | - | - | - | - |
| SugB | GH130 |  |  | + | - | - | + | - | + | - | - | - | - | - | | - | - | - | - |
| TreT |  |  | trehalose glycosyltransferring synthase | - | - | - | - | - | - | - | - | - | - | - | | - | - | - | - |

The symbol + indicates that the isolate produces the enzyme but symbol - indicates that the isolate does not produce the enzyme.


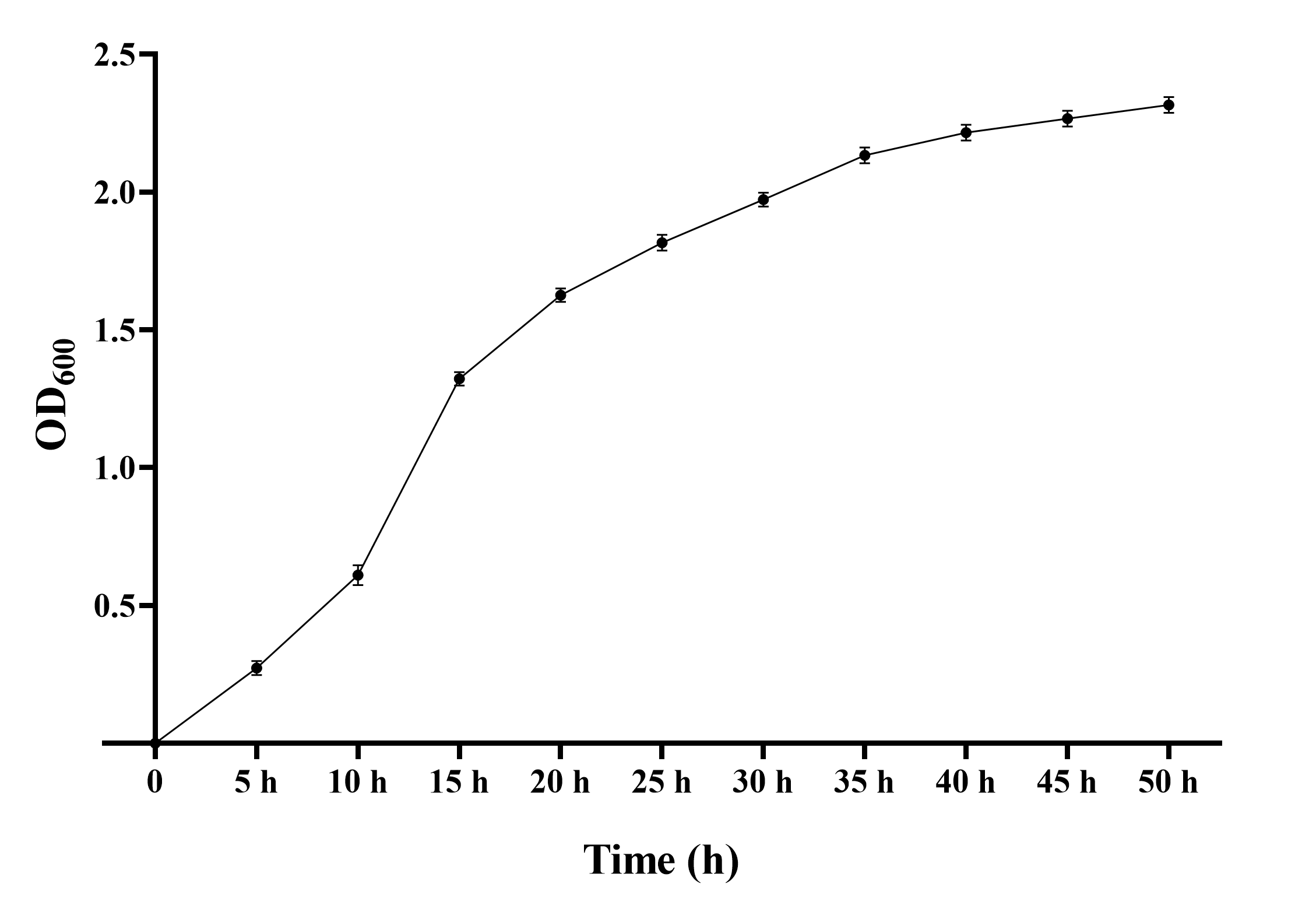


Figure S1 The growth curve of the W-6 strains at 15°C.
